# Clinical value of habitat radiomics within the 2-cm edema zone surrounding the postoperative residual cavity in predicting glioma recurrence

**DOI:** 10.3389/fonc.2026.1786939

**Published:** 2026-04-21

**Authors:** MengYu Cheng, Dong Bai, Wenjuan Liu, Jiawei Fan, Yuanzi Liang, Wenfei Li, Zhiqun Wang

**Affiliations:** 1 Department of Radiology, Aerospace Center Hospital, Beijing, China; 2 Department of Radiology, The First Hospital of Qinhuangdao, Qinhuangdao, China

**Keywords:** glioma, habitat radiomics, heterogeneity, nomogram, PFS prediction

## Abstract

**Objective:**

To assess the predictive value of radiomics from the 2-cm edema zone surrounding the postoperative residual cavity for progression-free survival (PFS), using habitat analysis based on multimodal magnetic resonance imaging (MRI) and integrating clinical data to construct a nomogram model.

**Methods:**

This retrospective study analyzed MRI and clinical data from 89 postoperative glioma patients. The 2-cm edema zone surrounding the postoperative residual cavity was defined as the region of interest (ROI), and habitat subregions were created using K-means clustering based on contrast-enhanced T1-weighted imaging (CE-T1WI) and apparent diffusion coefficient (ADC) sequences. Radiomic features were extracted from the ROI and each habitat subregion, followed by Least Absolute Shrinkage and Selection Operator (LASSO)-Cox selection to generate radiomic scores. Clinical, traditional radiomic, and high-risk habitat models were constructed, and the high-risk habitat nomogram was further developed and evaluated.

**Results:**

Four habitat subregions were identified. A total of 944 radiomic features were extracted from each subregion and the ROI; the most relevant features were used to generate radiomic scores. The high-risk habitat nomogram was constructed by combining clinical factors. The nomogram showed good calibration, with observed values closely matching predictions. In the validation cohort, the time-dependent AUCs for predicting 1-, 2-, and 3-year PFS were 0.813, 0.933, and 0.930, respectively. Compared with the clinical and traditional radiomic models, the high-risk habitat nomogram achieved a C-index of 0.916.

**Conclusion:**

The nomogram based on high-risk habitats in the 2-cm edema zone surrounding the postoperative residual cavity provides significant predictive value for PFS and aids in targeting postoperative radiotherapy.

## Introduction

Gliomas are the most common and aggressive primary brain tumors of the central nervous system, accounting for approximately 27% of all central nervous system tumors (1). Each year, around 100,000 individuals worldwide are diagnosed with glioma (2). Despite standard treatment, which includes surgical resection combined with adjuvant radiotherapy and chemotherapy (3), more than half of patients experience tumor recurrence within 7 to 10 months after surgery, with a median survival of 14 to 16 months (4). This poor prognosis is largely attributable to glioma heterogeneity, which is not limited to the contrast-enhancing tumor core observed on conventional MRI, but also includes the peritumoral edema region (5, 6). Studies have demonstrated that the peritumoral edema zone contains complex abnormal vasculature and infiltrative tumor cells, which are difficult to identify on preoperative conventional MRI or intraoperative fluorescence imaging, making complete resection challenging. Residual tumor cells are predominantly located within 2 cm of the resection cavity, leading to tumor recurrence in approximately 80% of cases within this area, reflecting the aggressiveness of gliomas. Therefore, early prediction of the heterogeneity surrounding the postoperative cavity is of significant clinical value for guiding individualized treatment (7).

Tumor heterogeneity reflects genetic differences and spatial variations in biological behavior, leading to the formation of tumor subregions known as “habitats” (8).Habitat radiomics can convert medical images into high-dimensional quantitative data that capture intratumoral heterogeneity (9, 10). Previous studies have primarily focused on using two-dimensional parameters to predict prognosis in glioma patients by defining the infiltrative tumor region (11, 12). Habitat imaging offers an alternative approach, capturing morphological diversity within habitats to noninvasively represent the heterogeneity of the tumor and surrounding infiltrative regions. This allows for the visualization of regions with similar heterogeneity and provides a rational basis for predicting patient prognosis (13, 14).

Magnetic resonance imaging (MRI) is extensively used for glioma diagnosis, treatment evaluation, and prognostic analysis (15). Previous studies have focused primarily on radiomics based on contrast-enhanced T1-weighted imaging (CE-T1WI), with limited investigation into the apparent diffusion coefficient (ADC) sequence (16–18). Therefore, this study aimed to construct a high-risk habitat predictive model based on CE-T1WI and ADC sequences, utilizing habitat analysis to identify heterogeneity in the 2-cm edema zone surrounding the postoperative residual cavity. The model was validated to provide an imaging foundation for individualized treatment of glioma patients.

## Materials and methods

### Study design

**Figure 1:**
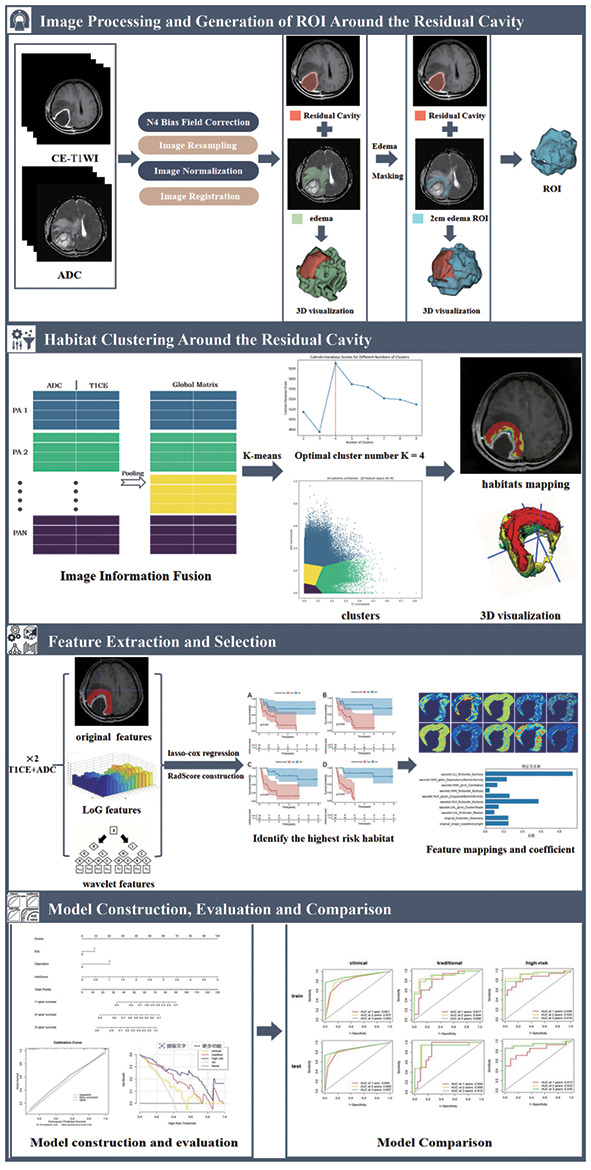
Overview of research workflow.

The research steps of this study are as follows: image acquisition, image preprocessing, generation of region of interest (ROI) around the residual cavity, habitat clustering around the residual cavity, feature extraction and selection, construction of a nomogram model, evaluation of the nomogram model, and comparative analysis of the three models, as shown in **Figure 1**.

This retrospective study was approved by the Ethics Committee of the First Hospital of Qinhuangdao (approval number: IRB No. 2023YY099). Due to the retrospective nature of the study, the requirement for informed consent was waived by the Ethics Committee, and the study was conducted in accordance with the Declaration of Helsinki. We retrospectively analyzed clinical and imaging data from patients treated between January 2016 and December 2020. Patients were randomly divided into a training cohort (n=50) and an independent validation cohort (n=39) using a computer-generated random sequence to ensure reproducibility.

Inclusion criteria were as follows: (1) complete clinical data and follow-up MRI images; (2) postoperative histopathological diagnosis of glioma; (3) basic normal function of major organs (heart, lungs, liver, kidneys); (4) MRI performed within one week after surgery, including complete conventional, contrast-enhanced, and ADC sequences, with image quality meeting evaluation standards. Exclusion criteria were: (1) patients who received other treatments before surgery; (2) multiple lesions; (3) incomplete imaging or clinical data, the presence of artifacts, or insufficient follow-up; (4) other malignancies or central nervous system diseases; (5) early postoperative death.

All included patients underwent follow-up MRI within one week postoperatively, which was used as baseline imaging data. During postoperative treatment, patients were followed until December 31, 2021, with follow-up MRI performed at 1, 3, and 6 months postoperatively, and every 6 months thereafter, following the “Guidelines for the Diagnosis and Treatment of Gliomas (2018 edition)” (19). The primary endpoint was PFS. The interval between the first postoperative MRI and tumor recurrence, progression, or last follow-up was defined as PFS, and the time from the first MRI to death or last follow-up was defined as OS. Recurrence was defined based on the Response Assessment in Neuro-Oncology (RANO) criteria (20, 21): (1) an increase in the sum of the perpendicular diameters of the enhancing lesions by ≥25% or an increase in the maximum diameter by >5 mm; (2) a significant increase in non-enhancing lesions on T2WI/FLAIR; (3) the appearance of new lesions.

### Image acquisition

All patients in the training and validation cohorts underwent postoperative MRI using a Siemens avanto 1.5T scanner equipped with an eight-channel head coil. Imaging sequences included axial T1-weighted imaging (T1WI), T2-weighted imaging (T2WI), fat-saturated T2 FLAIR, diffusion-weighted imaging (DWI; b=0, 1000), and axial contrast-enhanced T1WI. For all structural sequences (T1WI, T2WI, T2 FLAIR, and CE-T1WI), the slice thickness was 5 mm, with no gap, and a field of view (FOV) of 230 mm × 230 mm. Specific parameters were as follows: T1WI, repetition time (TR) 550 ms, echo time (TE) 9.04 ms; T2WI, TR 4400 ms, TE 107 ms; T2 FLAIR, TR 8000 ms, TE 120 ms; CE-T1WI, TR 600 ms, TE 7.2 ms; DWI, TR 3500 ms, TE 89 ms, matrix 256 × 256, slice thickness and FOV matched to structural sequences, b-values of 0 and 1000 s/mm², with a scan time of 68 s. The contrast agent used for T1WI was gadopentetate dimeglumine (China Beilu Pharmaceutical), administered at 0.2 mmol/kg, followed by a saline flush of 20 mL at 0.4 mL/s.

### Image processing and generation of ROI around the residual cavity

All imaging data were exported from the Picture Archiving and Communication System (PACS). The original DICOM(.dcm)files of the ADC and CE-T1WI sequences were imported into Anaconda 3 (Python 3.8.1, Jupyter Notebook 6.3.0) for preprocessing. First, N4 bias field correction was performed using the SimpleITK library (version 2.1.1) to minimize magnetic field inhomogeneities caused by the scanner, thereby homogenizing image brightness. Furthermore, to standardize the spatial resolution and minimize the effects of varying slice thicknesses, all corrected images were resampled to an isotropic voxel size of 1×1×1 mm³ using linear interpolation. Lastly, the ADC sequences were strictly co-registered to the CE-T1WI sequences.

The preprocessed images were then imported into 3DSlicer software (version 5.2.2; version 5.2.2; Surgical Planning Laboratory, Harvard University, Boston, MA, USA) for comparison of ADC and CE-T1WI sequences for each patient. Delineate the edema region and postoperative cavity, as well as the surrounding contrast-enhancing areas. To investigate the heterogeneity of the peritumoral edema zones around the residual cavities, we systematically expanded these initial residual cavity ROIs radially outward by 2 cm using custom automated Python scripts, and removed the excess edema tissue beyond the 2-cm boundary. The selection of a 2-cm margin was based on previous clinical and pathological evidence demonstrating that approximately 80% of glioma recurrences and occult microscopic infiltrations occur within this specific peritumoral zone (7, 22, 23). Subsequently, the generated 2-cm ROIs were imported into ITK-SNAP software (version 3.8; University of Pennsylvania Image Computing and Science Laboratory, Philadelphia, PA, USA)for minor manual adjustments strictly to exclude non-brain parenchyma areas extending into the skull base or ventricles.

To ensure the objectivity and robustness of the semi-automatic segmentation technique, two experienced radiologists independently delineated the areas without knowing the clinical and pathological information. In case of any uncertainty, a senior physician with 20 years of experience would make the final determination.

### Habitat clustering around the residual cavity

To ensure uniformity across patients, the image intensities of all voxels within the 2-cm ROIs were compiled into a global matrix after preprocessing. Prior to clustering, global intensity normalization was performed using min-max scaling derived exclusively from the training cohort voxels to avoid data leakage. Voxel-based K-means clustering was then applied using the unsupervised learning algorithm in Python. The optimal number of clusters was determined to be 4 based on the Calinski-Harabasz score calculated solely on the training data (tested from 2 to 9, using the maximum inflection point of the evaluation curve). The learned centroids were subsequently applied to assign habitat labels in the validation cohort.

### Feature extraction and selection

In accordance with the Imaging Biomarker Standardization Initiative (IBSI) guidelines, radiomic feature extraction was performed using the Pyradiomics package (version 3.0.1; Artificial Intelligence in Medicine Program, Harvard Medical School, Boston, MA, USA) (24). Radiomic features were systematically extracted from both the global ROI and each spatial habitat (ROI_habitat_). Ultimately, 944 features were extracted per region, comprising original first-order and shape features, Laplacian of Gaussian (LoG) features, and wavelet-transformed features. To ensure feature robustness against manual boundary variations, the traditional radiomic features extracted from the global 2-cm ROI were evaluated, and only those with an intraclass correlation coefficient (ICC) ≥ 0.75 were retained.

Missing values were imputed using the k-nearest neighbor algorithm, and Z-score normalization was applied for data standardization. Following a preliminary screening using the Mann-Whitney U test, the retained traditional features and the standardized habitat features were incorporated into a LASSO-Cox regression model. The optimal penalty parameter (λ) was determined via 10-fold cross-validation to select the most valuable prognostic features. Radiomic scores (RadScores) were then calculated using the glmnet package in R software (version 4.1.3; R Foundation for Statistical Computing, Vienna, Austria) (25). Patients were categorized into high- and low-risk groups based on the optimal RadScore cutoffs determined by X-tile software(version 3.6.1; Yale University, New Haven, CT, USA). Lastly, Kaplan-Meier survival analysis was performed to compare differences between the high- and low-risk groups, applying the same coefficients and cutoffs to the validation cohort.

### High-risk habitat identification and model construction

High-risk habitats were identified based on the RadScores extracted from each habitat (ROI_habitat_). Diagnostic efficacy was evaluated through AUC, sensitivity, and specificity analyses. Kaplan-Meier survival analysis was used to determine statistical differences between high- and low-risk groups. Three models were constructed: a clinical model, a traditional radiomic model, and a high-risk habitat model. The clinical model was constructed by incorporating significant clinical and imaging features identified through univariate Cox analysis, followed by multivariate regression using Akaike information criterion (AIC) to retain the optimal features. The traditional radiomic model included the global RadScores, and the high-risk habitat model included the RadScores from the selected high-risk habitat subregions.

### Statistical analysis

Continuous variables were compared using the Wilcoxon rank sum test or Welch Two Sample t-test. Categorical variables were compared using Fisher’s exact test or Pearson’s chi-square test. Univariate and multivariate Cox regression analysis (95% confidence interval) were used to investigate the independent clinical and imaging risk factors affecting the postoperative recurrence of glioma patients. ROC analysis accounting for right censoring was performed to evaluate the discriminatory ability of the models at 1, 2, and 3 years for PFS. All radiomics analyses were conducted using R (version 4.1.3), Anaconda 3 (Python 3.8.1), and IBM SPSS (version 26; IBM Corp., Armonk, NY, USA). The predictive performance of each habitat was evaluated using the AUC of ROC curves. K-M survival curves were plotted to compare survival differences between high- and low-risk RadScore groups. A nomogram was constructed using high-risk habitat RadScores and clinical factors, and predictive performance was evaluated using the concordance index (C-index) and hazard ratio (HR). Calibration curves were used to evaluate the fit of the nomogram model. Decision curve analysis (DCA) was used to assess the net clinical benefit of each model. The P-value < 0.05 was considered statistically significant.

## Results

### Patient clinical characteristics

A total of 89 glioma patients, comprising 43 males (48.3%) and 46 females (51.7%), were included in this study. Patients were randomly divided into a training cohort (n=50) and an independent validation cohort (n=39).In the validation cohort (n = 39), 20 patients experienced disease progression (51.3%) while 19 patients did not (48.7%); the median age was 51 years (range, 21–76 years), comprising 20 males (51.3%) and 19 females (48.7%). There were no significant differences in clinical characteristics between the two cohorts (P > 0.05). Detailed clinical and imaging characteristics are provided in **Table 1**.

**Table 1:** Clinical and imaging characteristics of patients in the training and validation cohorts.

Characteristic	Training,N=50^1^	Validation, N=39^1^	p-value^2^
Age	51 (42, 62)	51 (42, 63)	0.611
gender(male, %)	23(46.0)	20(51.3)	0.621
Involved functional area (n, %)	22(44.0)	15(38.5)	0.599
Maximum postoperative cavity diameter (n, %)			0.826
d<5cm	33(66.0)	24(61.5)	
d≥5cm	17(34.0)	15(38.5)	
Minimum postoperative cavity diameter			0.885
d<3cm	30(60.0)	22(56.4)	
d≥3cm	20(40.0)	17(43.6)	
Surgical resection (n, %)			0.639
Subtotal resection	23(46.0)	16(41.0)	
Gross total resection	27(54.0)	23(59.0)	
Pathological grade (n, %)			0.787
II	18(36.0)	15(38.5)	
III	12(24.0)	7(17.9)	
IV	20(40.0)	17(43.6)	
Postoperative radiotherapy and chemotherapy (n, %)	40(80.0)	30(76.9)	0.725

^1^n (%).

^2^Wilcoxon rank sum test; Welch Two Sample t-test; Pearson's Chi-squared test; Fisher's exact test.

### Habitat clustering and high-risk subregion identification

**Figure 2:**
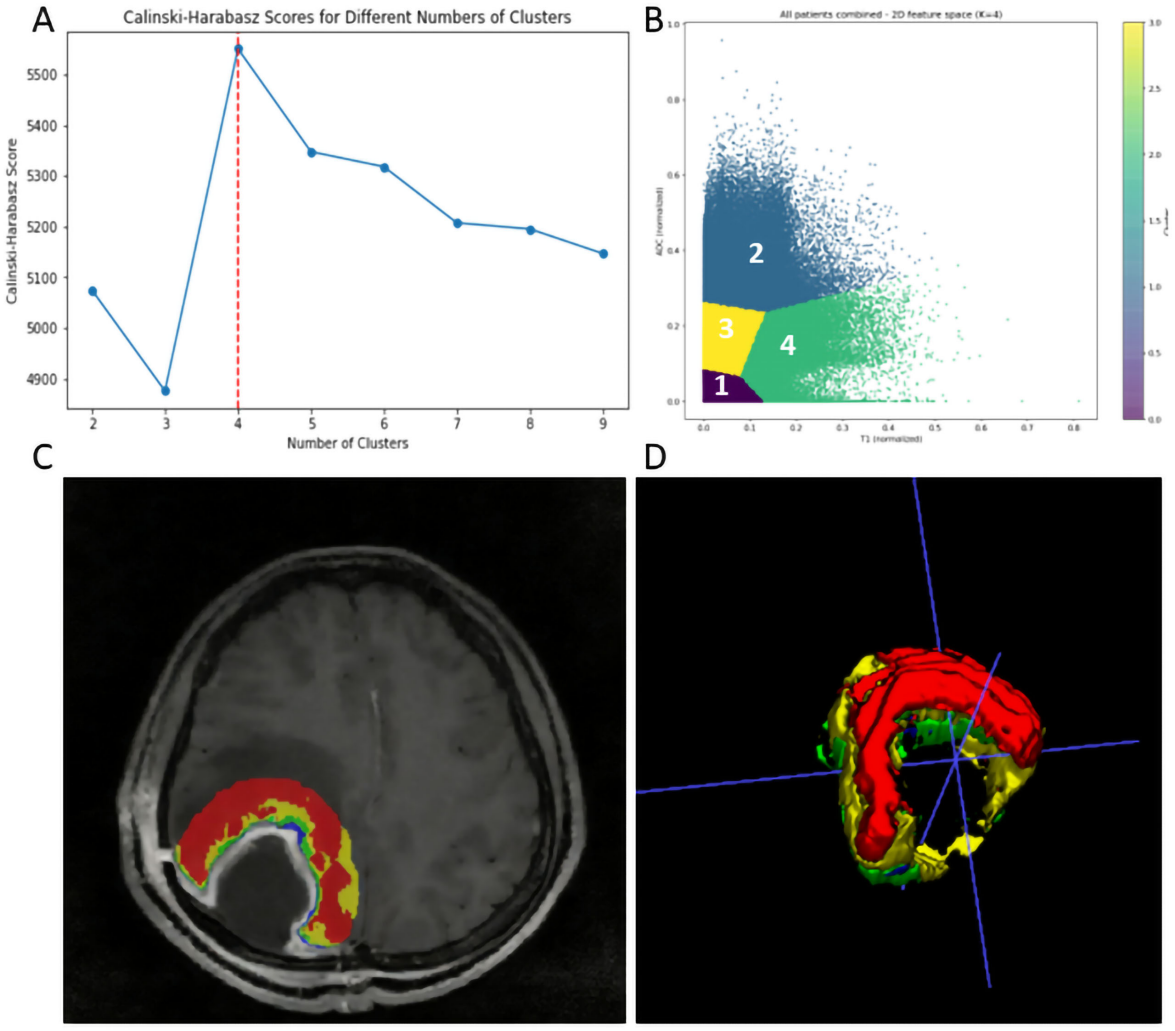
The K-meaning clustering method was used to cluster and visualize ROI, and the algorithm was used to calculate the optimal number of clusters. When K = 4, the maximum inflection point **(A)** appeared, and the four items of clustered voxels were visualized in the form of scatter plot **(B)** to generate habitat subarea map **(C)** and 3D visualization **(D)**.

To investigate the heterogeneity of the peritumoral edema zone around the residual cavity, K-means clustering was performed on the global intensity matrix of the 2-cm edema zone surrounding the postoperative residual cavity. The optimal number of clusters was determined to be k = 4 based on the maximum inflection point of the Calinski-Harabasz score (**Figure 2A**). Consequently, four distinct habitat subregions were delineated (**Figures 2B, D**).

As visualized in the normalized 2D feature space scatter plot (**Figure 2B**), the four habitats were clearly separated along the CE-T1WI (x-axis) and ADC (y-axis) axes, corresponding to distinct biological microenvironments within the peritumoral edema. Habitat 1 (lowest ADC) represented necrotic or hemorrhagic foci. Habitat 2 (highest ADC) indicated pure vasogenic edema or nonviable tissue with unrestricted water diffusion. Habitat 3 corresponded to mildly infiltrative or fibrotic regions. Habitat 4 represented high-cellularity infiltrative tumor regions with restricted diffusion. (**Figures 2C, D**) show the clustering map and 3D visualization generated after clustering.

Quantitative characteristics of the four habitats in the validation cohort (n=39) are summarized in **Supplementary Table 3**. Habitat 4 occupied the largest mean volume proportion with a distinctly lower ADC compared with Habitat 2, supporting its biological role as the dominant high-cellularity infiltrative tumor component. The same K-means clustering algorithm was applied to the independent validation cohort.

**Figure 3:**
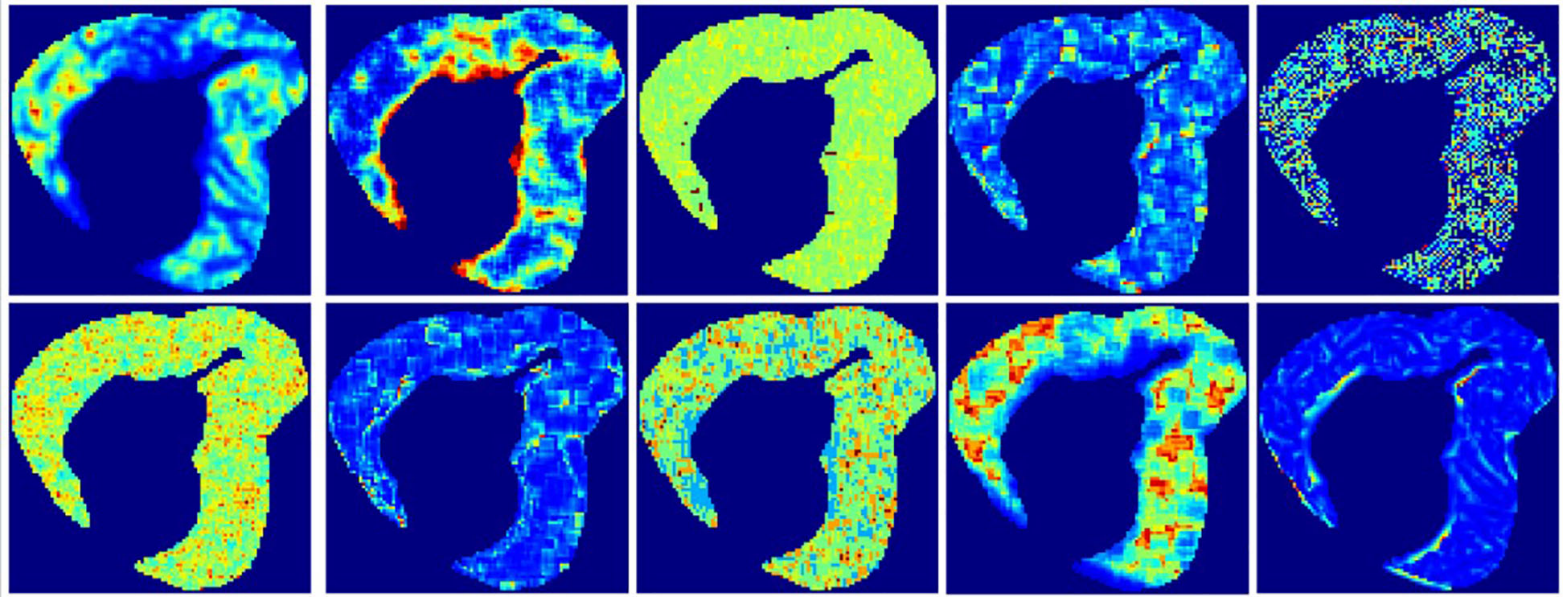
The 1–10 feature mappings selected for high-risk habitats correspond to: original_firstorder_Skewness, original_shape_LeastAxisLength, wavelet. lhl_glcm_ClusterShade, wavelet-HHH_firstorder_ Kurtosis, wavelet-HHH_glcm_Correlation, wavelet-HHH_gldm_DependenceNonUniformity, wavelet-HLH_firstorder_Kurtosis HLH_firstorder_Kurtosis, wavelet-HLH_ glszm_Gray LevelNon-Uniformity, wavelet-LHL_firstorder_Median, wavelet-LLL_firstorder_Kurtosis.

**Figure 4:**
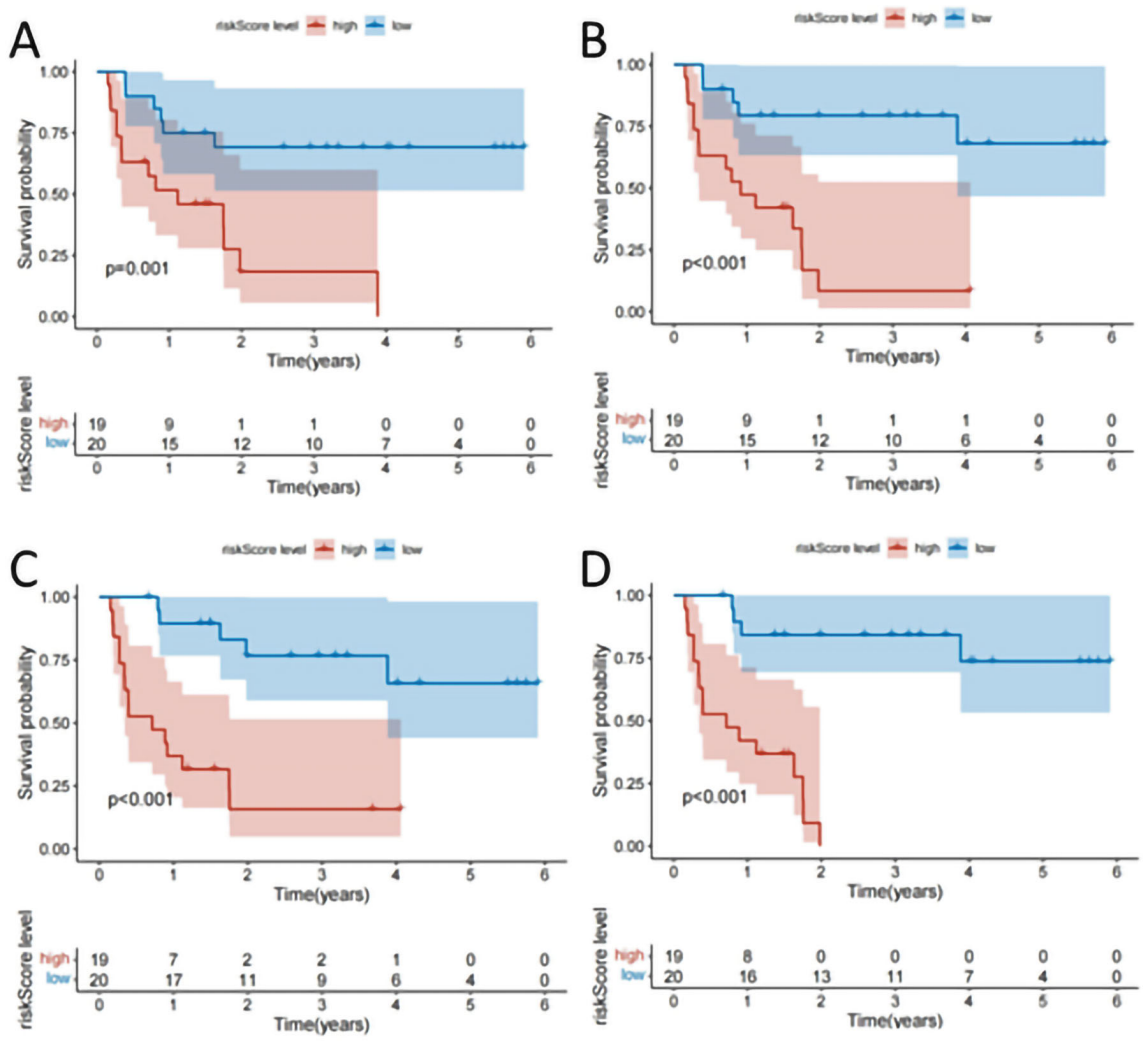
**(A–D)** The K-M survival curves of habitat subareas 1–4 of the verification cohort.

**Figure 5:**
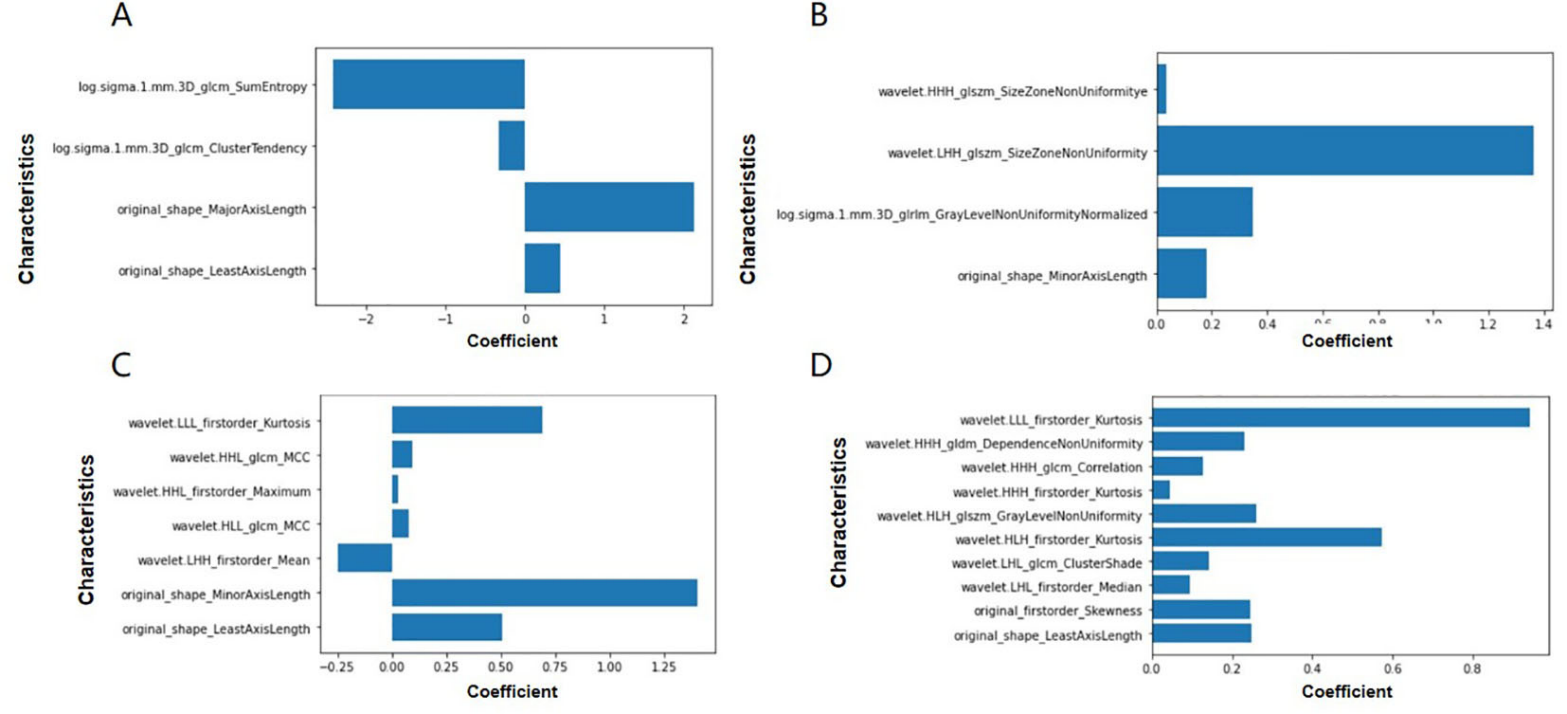
**(A–D)** Characteristics and corresponding coefficients of four habitat subareas.

Following clustering, 944 radiomic features were extracted from each subregion, including 107 original features, 93 Laplacian of Gaussian (LoG) features, and 744 wavelet-based features. LASSO-Cox regression was applied to select the most valuable features and calculate the RadScores for each habitat (**Figure 3**). ROC and Kaplan-Meier (K-M) survival analyses were conducted to evaluate the predictive performance of each subregion (**Table 2**, **Supplementary Figure S2**, **Figure 4**, **Supplementary Figure S2**). The K-M survival curves revealed that Habitat 4 demonstrated the most statistically significant difference in prognosis among all subregions (**Figure 4D**, **Supplementary Figure S2D**), successfully identifying it as the high-risk habitat for PFS (**Figure 5** details the optimal features and corresponding coefficients for each subregion).

**Table 2:** Habitat clustering analysis in the validation cohort.

Subregion	Validation group		
	C-index	HR(95%CI)	p-value
Habitat Subregion 1	0.813	0.07(0.65-0.92)	<0.0001
Habitat Subregion 2	0.834	0.07(0.68-0.93)	<0.0001
Habitat Subregion 3	0.845	0.07(0.69-0.94)	<0.0001
Habitat Subregion 4	**0.861**	0.08(0.71-0.95)	<0.0001

HR: Hazard Ratio;.

Data in parenthesis are 95% confidence intervals;.

Black bold fonts represent the best performing habitat subregions.

### Construction of prognostic models

Three predictive models were independently constructed to evaluate progression-free survival (PFS):

#### Clinical model

Clinical and imaging factors, including pathological grade and age (both analyzed as categorical and continuous variables respectively), were incorporated into univariate and multivariate Cox regression analyses (**Supplementary Table 1**). Pathological grade was significantly associated with PFS in the univariate analysis but lost statistical significance in the multivariable model after adjustment for surgery type and functional area involvement. In contrast, surgery type and involvement of functional areas remained independent prognostic factors. These variables were retained to construct the clinical model.

#### Traditional radiomic model

The reproducibility of the 944 traditional radiomic features extracted from the global 2-cm ROI was first evaluated. Features with an intraclass correlation coefficient (ICC) < 0.75 were considered unstable and excluded. The remaining robust features underwent preliminary screening and LASSO-Cox regression to select significant features and compute a global RadScore. ROC curves were generated for both the training and validation cohorts based on RadScore, with an AUC of 0.821 (95% CI, 0.665-0.925, P < 0.001), sensitivity of 90.0%, and specificity of 78.9% in the validation cohort. This RadScore was then combined with independent clinical risk factors to establish the traditional radiomic model.

#### High-risk habitat nomogram model

**Figure 6:**
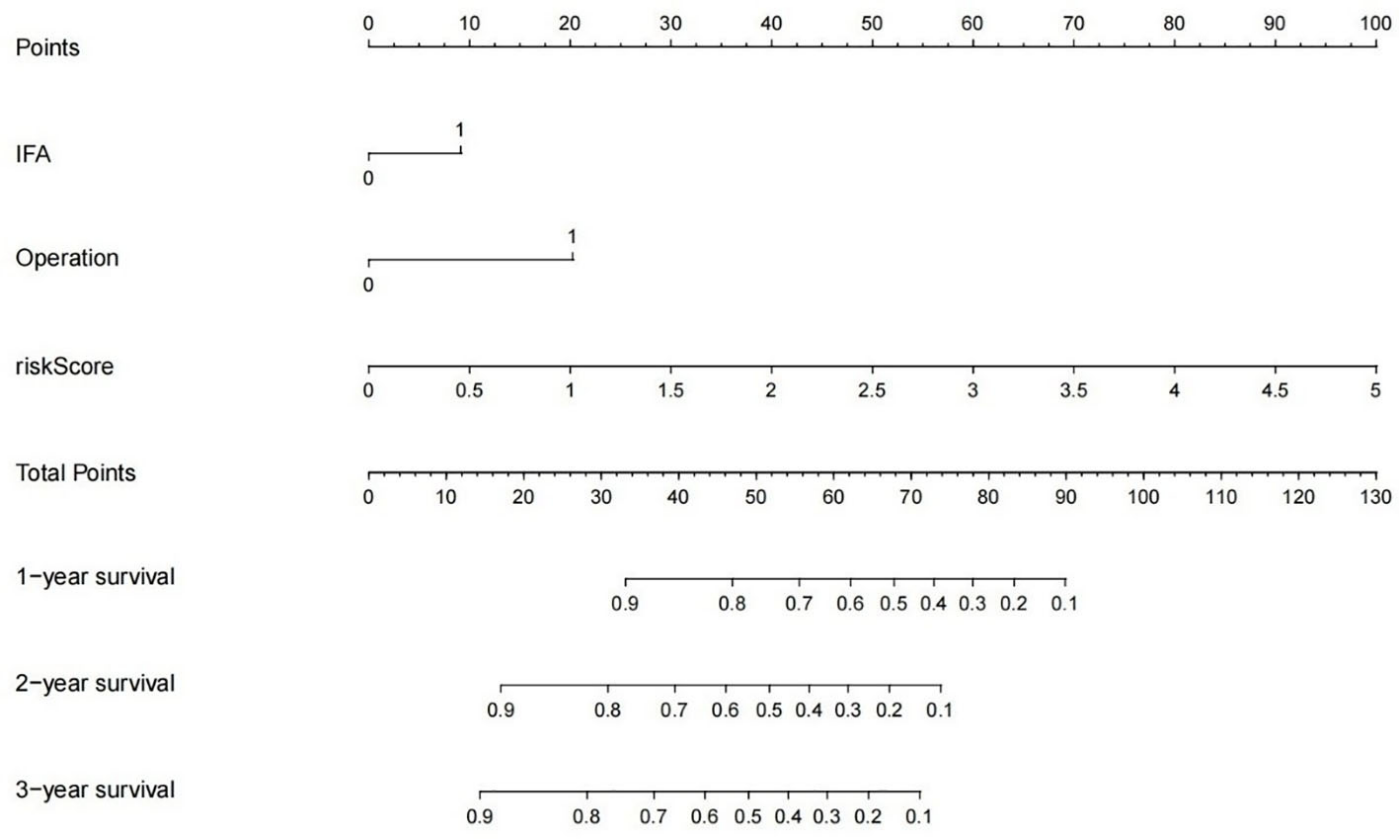
High risk habitat nomogram. The nematic model showed that IFA, Operation, and riskscore of optimal high-risk habitat subregions were independent risk factors affecting postoperative prognosis of glioma patients, and the cumulative survival rate of postoperative patients could be estimated by 1/2/3 years according to the score of each index.(IFA:Involved functional area; Operation:Subtotal resection or Gross total resection).

The RadScores derived strictly from the identified high-risk habitat (Habitat 4) were integrated with the significant clinical factors to generate a highly visual high-risk habitat nomogram (**Figure 6**) for individualized PFS prediction.

### Evaluation and comparison of the models

To comprehensively assess the performance of the three models in predicting PFS, we evaluated them from three dimensions: discrimination, calibration, and clinical utility.

#### Discrimination

**Figure 7:**
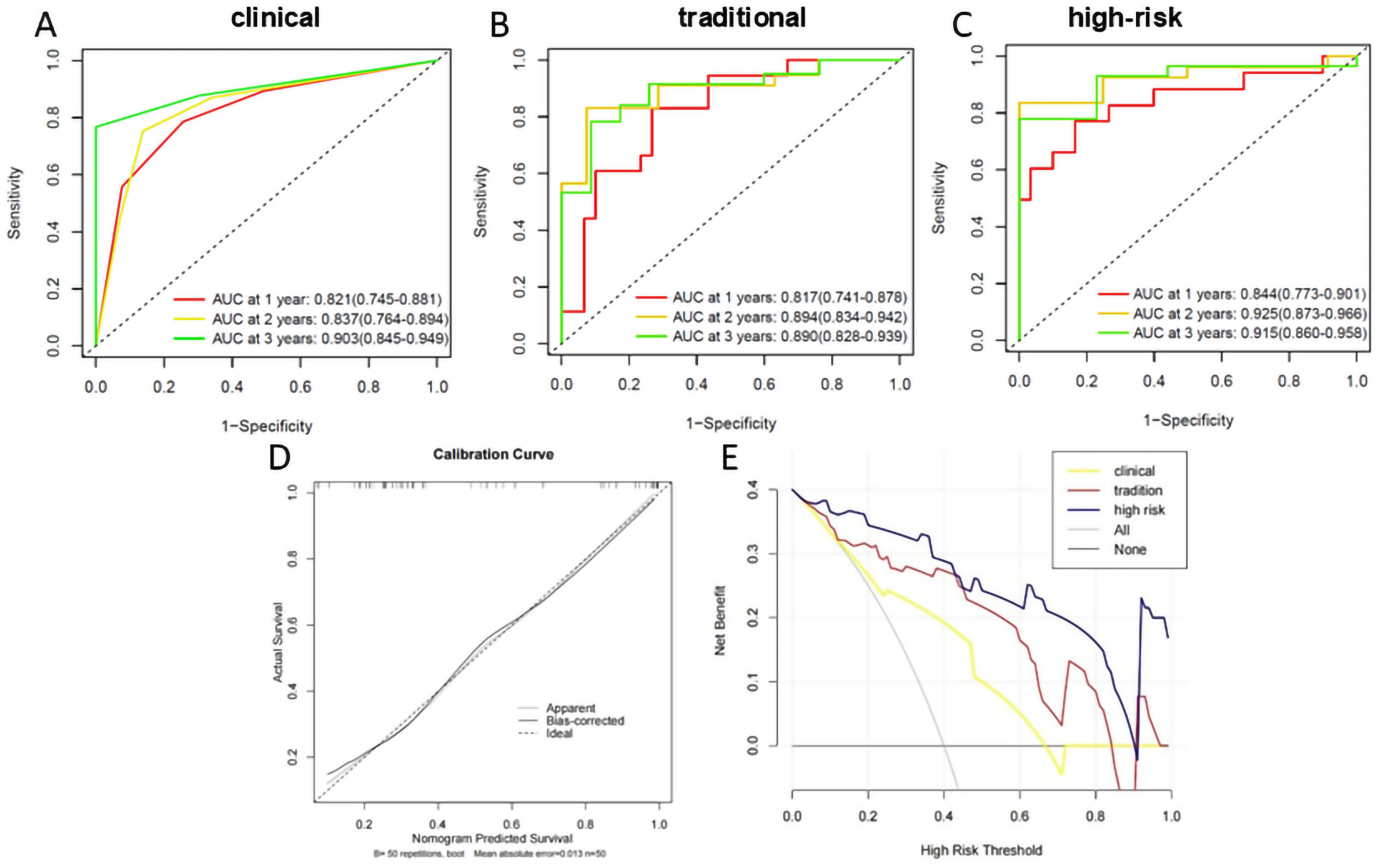
Time-dependent receiver operating characteristic (ROC) curves accounting for right censoring for predicting 1-, 2-, and 3-year progression-free survival (PFS) in the validation cohort. **(A)** Clinical model, **(B)** traditional radiomic model, **(C)** high-risk habitat nomogram. **(D)** Calibration curve of the high-risk habitat nomogram in the validation cohort. **(E)** DCA comparing the net benefit of the three models.

The high-risk habitat nomogram demonstrated favorable performance, achieving the highest C-index of 0.916 in the validation cohort (**Table 3**). The time-dependent AUCs for predicting 1-, 2-, and 3-year PFS in the validation cohort were 0.813, 0.933, and 0.930, respectively (**Figure 7C**). These values outperformed both the traditional radiomic model and the clinical model (**Figures 7A, B**).

**Table 3:** Prognostic performance of clinical, conventional radiomics and high-risk habitat models(Validation cohort).

Model	Validation cohort	
	C-index	p-value
Clinical Model	0.843	0.015
Conventional Radiomics Model	0.885	0.005
High-Risk Habitat Model	**0.916**	0.007

Bold values indicate the best-performing model.

#### Calibration

The Transparent Reporting of a multivariable prediction model for Individual Prognosis Or Diagnosis (TRIPOD) guidelines (26)recommend reporting model calibration performance, as poor calibration may reduce the clinical utility of the model (27). Calibration curves were generated specifically to evaluate the predictive accuracy of the high-risk habitat nomogram. The curves demonstrated excellent agreement between the nomogram-predicted probabilities of 1-, 2-, and 3-year PFS and the actual clinical observations in both the validation cohort, with minimal mean absolute errors (MAE: 0.032) (**Figure 7D**).

#### Clinical utility

Decision curve analysis (DCA) helps evaluate treatment strategies and suggests treatment for patients estimated to be at “high risk.” Generally, the strategy with the highest net benefit at a specific threshold probability has the highest clinical utility (28). In this study, DCA curves were generated for the clinical model, traditional radiomic model, and high-risk habitat model in the validation cohort (**Figure 7E**). Across the majority of the threshold probabilities, the high-risk habitat nomogram consistently provided a higher net clinical benefit for predicting progression-free survival (PFS) compared with both the traditional radiomic and clinical models, as well as the “treat-all” or “treat-none” strategies. These results indicate that the high-risk habitat nomogram offers superior clinical utility in guiding postoperative decision-making.

## Discussion

This study attempted to use habitat radiomics to cluster subregions of the 2-cm edema zone surrounding the postoperative residual cavity after glioma surgery, identify high-risk habitats, and integrate clinical models to establish a high-risk habitat nomogram closely related to PFS. The constructed nomogram model demonstrated high predictive value. By comparing different models, the high-risk habitat model exhibited significantly better predictive performance than the clinical and traditional radiomic models. The nomogram can provide valuable prognostic information to clinicians for postoperative treatment, assist in formulating personalized treatment plans for patients.

Three models were constructed in this study: the clinical model, the traditional radiomic model, and the high-risk habitat model. In constructing the clinical model, Cox univariate and multivariate regression analyses identified surgical resection type and functional area involvement as independent prognostic factors for postoperative glioma outcomes. Surgical resection type is a critical prognostic factor for glioma, with literature reporting that gross total resection leads to significantly better overall survival (OS) and PFS compared to subtotal resection (29, 30). This finding is consistent with previous research. The tumor location in gliomas often leads to various clinical symptoms, and during surgery, the involvement of functional areas that cause clinical symptoms inevitably leads to adverse effects on prognosis (10, 31). This conclusion further emphasizes the importance of tumor location, and our study similarly found that functional area involvement significantly influences patient prognosis. Pathological grade has been consistently reported as an independent prognostic factor in multiple studies on glioma prognosis (29, 32). In the present study, however, pathological grade was significant in the univariate Cox analysis but lost statistical significance in the multivariable model after adjustment for surgery type and functional area involvement (**Supplementary Table 1**). This discrepancy likely reflects the limited statistical power due to the relatively small sample size (total n=89) and the high proportion of high-grade gliomas (WHO grade III/IV accounted for 64.0% in the validation cohort), which may have reduced the ability to detect an independent effect. It is also possible that, in the postoperative setting, the influence of pathological grade is partially mediated through surgical resection extent and functional area involvement. Future studies with larger, more balanced cohorts are warranted to further clarify the independent prognostic value of pathological grade after glioma surgery. Previous studies (29, 33)have shown that age is an independent prognostic factor for glioma patients, but our findings differed, possibly because of the wide age range and the predominance of high-grade gliomas in this cohort. Age was significant in the univariate analysis but lost significance in the multivariate model after adjustment for surgery type and functional area involvement (**Table 2**). Several studies (29, 34)have confirmed the application value of clinical models in predicting PFS. Clinical models are relatively simple and easy to understand, but they only perform well in certain populations or datasets and may not generalize well to external datasets, resulting in reduced stability and accuracy.

With advancements in computing and artificial intelligence, radiomic analysis has made significant progress in establishing predictive models for diagnosis, prognosis, and treatment response by extracting quantitative features such as intensity, texture, and geometry from the entire tumor imaging dataset (35–37). Radiomics can effectively reflect the intrinsic state of tumors and the tumor microenvironment. Our findings and previous studies (38–40)have established glioma predictive models based on multimodal MRI radiomics, demonstrating that radiomic models based on ADC and CE-T1WI sequences have superior prognostic accuracy compared to clinical models and have been independently validated. However, past studies were based on baseline imaging and did not consider heterogeneity in the peri-cavity region after glioma surgery. Previous studies (34)have confirmed that the mean ADC value in the peri-cavity region is a risk factor for glioma recurrence postoperatively [HR = 0.95 (0.91-0.99)], validating the importance of peri-cavity heterogeneity in postoperative prognosis. However, previous studies (40, 41)were based on two-dimensional quantitative parameters and did not consider global radiomic features. Our study, using a global radiomic model of the peri-cavity region, confirmed the significance of peri-cavity heterogeneity for prognosis, with improved accuracy compared to clinical models, thereby overcoming some of the limitations of clinical models. However, it does not provide additional dimensional information about the internal heterogeneity of the tumor or the potential mechanisms of tumor growth, diffusion, and treatment resistance.

Compared to radiomics of the glioma core or peri-cavity edema region (42), habitat imaging allows noninvasive quantification of subregions more closely related to tumor growth or invasiveness. In the present study, the four habitats identified in the 2-cm peri-cavity edema zone biologically represent distinct microenvironments within the postoperative residual cavity. Habitat 2 (highest ADC) corresponds to pure vasogenic edema or nonviable tissue with unrestricted water diffusion. Habitat 4 (lower ADC) captures high-cellularity infiltrative tumor regions with restricted diffusion—the dominant component responsible for local recurrence. Habitat 1 represents necrotic or hemorrhagic foci, while Habitat 3 indicates mildly infiltrative or fibrotic transition zones.

This biological interpretation is supported by imaging-pathologic correlation studies. Sun et al. (43)demonstrated using identical T1CE-ADC voxel-wise K-means clustering that low-ADC habitats correspond histologically to residual infiltrative tumor cells rather than pure edema. Our findings extend this by focusing exclusively on the 2 cm edema zone around the postoperative residual cavity, where Habitat 4 occupied the largest per-patient volume (38.28 ± 25.27%) with a distinctly lower ADC (1.048 ± 0.092 ×10^-^³ mm²/s) compared with the high-ADC edema region (Habitat 2). These quantitative differences, Bailo et al. (31) and Dextraze (44) further support that habitat subregions can accurately reflect biological information with similar pathological and molecular heterogeneity.

In this study, we compared the three models and found that the high-risk habitat model showed the best predictive performance, with numerically improved accuracy (C-index = 0.916 in the validation cohort) compared to clinical and radiomic models. Therefore, we constructed a nomogram model based on the high-risk habitat model combined with clinical features to predict postoperative outcomes in glioma patients. The nomogram demonstrated the highest predictive performance at 1, 2,and 3 years postoperatively, showing good stability.

Habitat imaging technology not only allows for the noninvasive identification of tumor and peri-cavity microenvironment heterogeneity, establishing a more precise predictive model (45–47)to improve diagnostic accuracy, but also visualizes features with similar biological behavior, enabling the monitoring of tumor response to treatment and timely adjustment of treatment strategies. The study by Park et al. (48) demonstrated that pathological heterogeneity can be quantified through noninvasive habitat imaging. The study used multiparametric MRI to establish spatial habitats and evaluated the temporal changes of these habitats in relation to PFS in GBM patients after concurrent chemoradiotherapy. In our study, we could clearly observe changes in tumor heterogeneity before and after chemoradiotherapy. Similar findings were reported by Kim et al. (49). Traditional radiomic models cannot visualize tumor heterogeneity and have limited interpretability. In this study, habitat imaging was used to visualize the 2−cm edema zone around the postoperative residual cavity, which is expected to provide a more accurate theoretical basis for the precise delineation of radiotherapy target volumes.

However, there are several limitations to this study. First, as a retrospective analysis, there is a potential for selection bias, and prospective studies are needed to further validate the model’s effectiveness. Second, this was a single-center study with a relatively small sample size (Total n=89). For high-dimensional habitat radiomics modeling, such limited cohorts increase the risk of overfitting and may compromise generalizability and stability. Moreover, the absence of external multi-center validation limits the broader applicability of the high-risk habitat nomogram. Future prospective multi-center studies with larger cohorts are warranted to confirm its robustness across different patient populations and scanner platforms.

## Conclusion

In conclusion, the high-risk habitat model constructed using habitat imaging is capable of predicting PFS in glioma patients and outperforms traditional radiomics and clinical models. The model demonstrates high AUC and stable performance, visualizing the heterogeneity in the 2 cm peri-cavity edema zone after glioma surgery, providing clinicians with the necessary information to create individualized treatment plans.

## Data Availability

The datasets presented in this article are not readily available because The datasets generated and analyzed during the current study are not publicly available due to institutional data privacy policies. Requests to access the datasets should be directed to cmy625729@163.com.

## Supplementary material

Supplementary Figure 1 Time-dependent receiver operating characteristic (ROC) curves accounting for right censoring for predicting 1-, 2-, and 3-year progression-free survival (PFS) in the Training cohort. **(A)** Clinical model, **(B)** traditional radiomic model, **(C)** high-risk habitat nomogram.**(D)** Calibration curve of the high-risk habitat nomogram in the Training cohort.**(E)** DCA comparing the net benefit of the three models.

Supplementary Figure 2 **(A–D)** The K-M survival curves of habitat subareas 1–4 of the training cohort.

Supplementary Table 1 Univariable and Multivariable Cox regression analysis of clinical and imaging risk factors.

Supplementary Table 2 Habitat clustering analysis in the training cohort.

Supplementary Table 3 Quantitative characteristics of the four habitat subregions in the Validation cohort (n=39).

Supplementary Table 4 Prognostic performance of clinical, conventional radiomics and high-risk habitat models(Training cohort).
