# Supplementary figures and images for: Clinical value of habitat radiomics within the 2-cm edema zone surrounding the postoperative residual cavity in predicting glioma recurrence
Source: Front Oncol. 2026 Apr 21;16:1786939. doi: 10.3389/fonc.2026.1786939 (PMC13138962; doi:10.3389/fonc.2026.1786939)

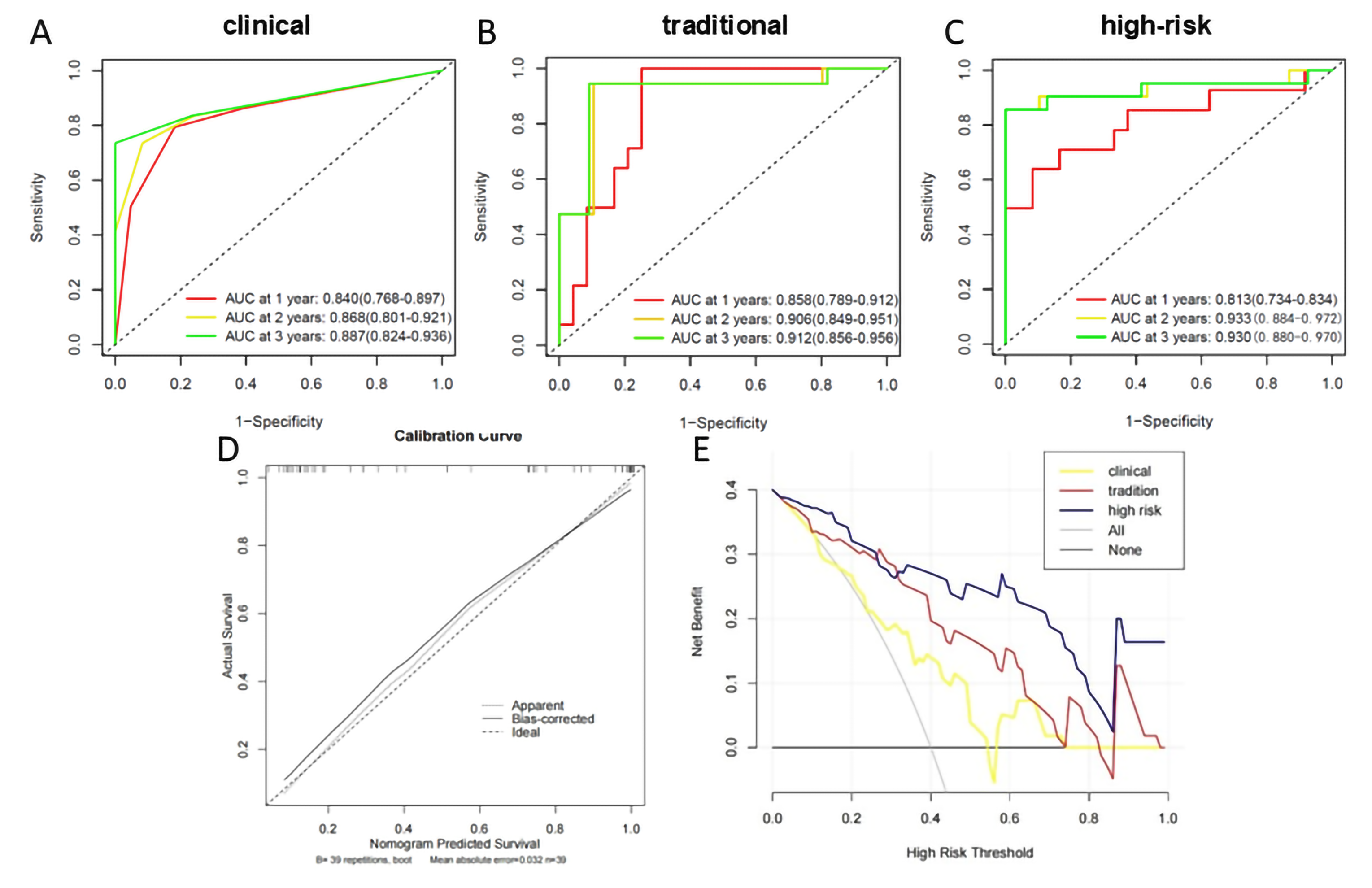

Supplement: Supplementary Figure 1 — Time-dependent receiver operating characteristic (ROC) curves accounting for right censoring for predicting 1-, 2-, and 3-year progression-free survival (PFS) in the Training cohort. (A) Clinical model, (B) traditional radiomic model, (C) high-risk habitat nomogram.(D) Calibration curve of the high-risk habitat nomogram in the Training cohort.(E) DCA comparing the net benefit of the three models. [file Image1.tif]

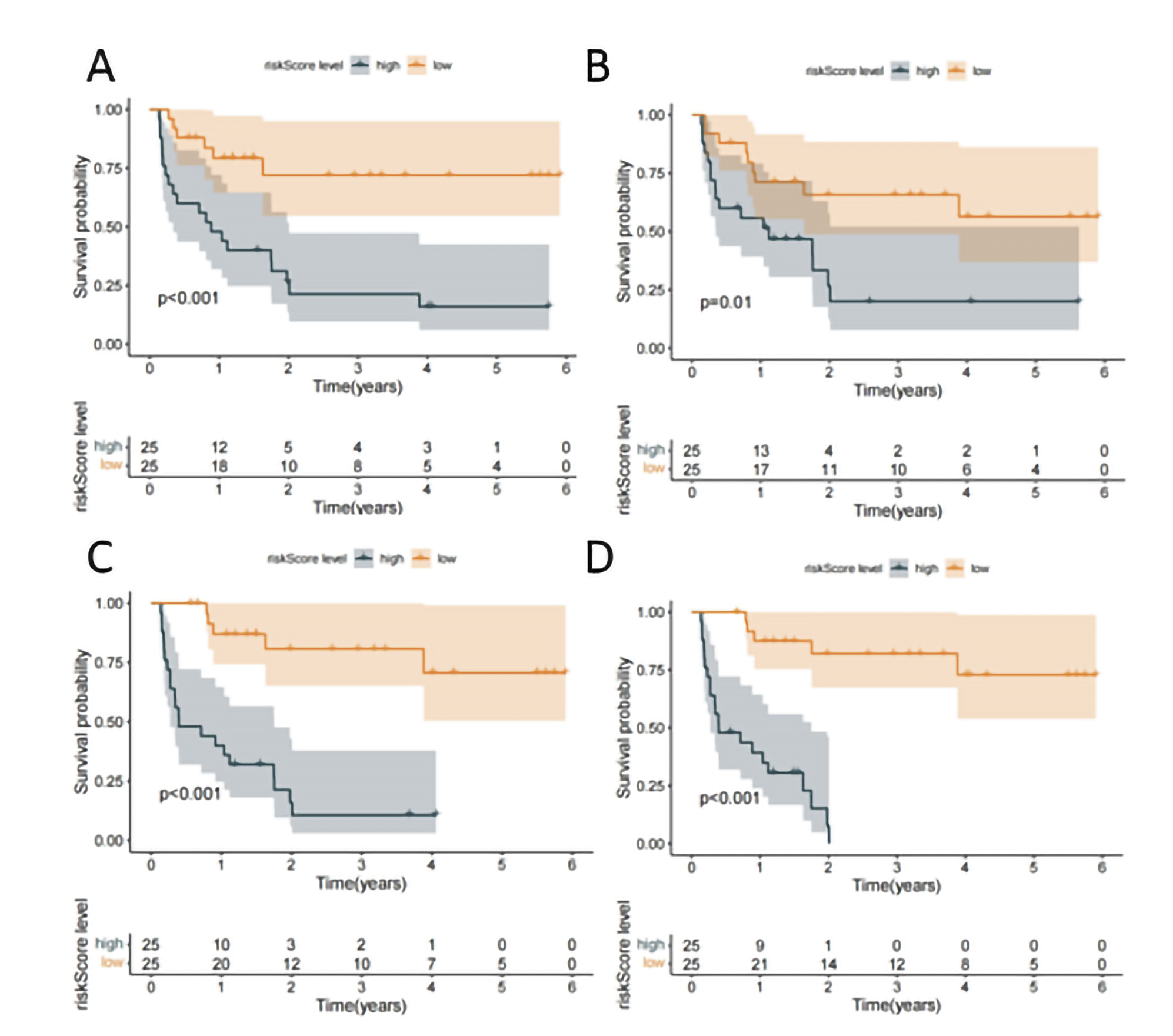

Supplement: Supplementary Figure 2 — (A–D) The K-M survival curves of habitat subareas 1–4 of the training cohort. [file Image2.tif]
