# Supplementary material for: Clinical value of habitat radiomics within the 2-cm edema zone surrounding the postoperative residual cavity in predicting glioma recurrence
Source: Front Oncol. 2026 Apr 21;16:1786939. doi: 10.3389/fonc.2026.1786939 (PMC13138962; doi:10.3389/fonc.2026.1786939)
Supplement: Supplementary Table 1 — Univariable and Multivariable Cox regression analysis of clinical and imaging risk factors. [file Table1.docx]

Table S1. Univariable and Multivariable Cox regression analysis of clinical and imaging risk factors

| Characteristic | HR | p-value | Adjusted HR* | p-value |
| --- | --- | --- | --- | --- |
| Age | 1.04 (1.01-1.08) | 0.006* | 1.02(0.99-1.06) | 0.285 |
| Gender | 1.18(0.55-2.54) | 0.678 |  |  |
| Involved functional area | 3.126(1.413-6.914) | 0.005* | 3.17(1.25- 8.02) | 0.015* |
| Maximum postoperative cavity diameter | 1.139(0.924-1.403) | 0.222 |  |  |
| Minimum postoperative cavity diameter | 1.231(0.854-1.775) | 0.266 |  |  |
| Surgical resection | 7.100(2.771-18.191) | <0.001* | 3.57(1.15- 11.05) | 0.027* |
| Pathological grade | 2.809(1.642-4.806) | <0.001* | 3.48(0.84-14.40) | 0.085 |
| Postoperative radiotherapy and chemotherapy | 0.925(0.371-2.307) | 0.868 |  |  |

Note: * Statistically significant (P<0.05);

HR: Hazard Ratio;

* Variables with *P* < .10 in the univariable analysis were included in the multivariable model for independent predictors.

Table S2. Habitat clustering analysis in the training cohort

| Subregion | Training cohort | | |
| --- | --- | --- | --- |
|  | C-index | HR(95%CI) | p-value |
| Habitat Subregion 1 | 0.845 | 3.50 (2.04-6.01) | ＜0.0001 |
| Habitat Subregion 2 | 0.806 | 3.66(2.05-6.53) | ＜0.0001 |
| Habitat Subregion 3 | 0.873 | 5.97(2.88-12.35) | ＜0.0001 |
| Habitat Subregion 4 | 0.885 | 4.17 (2.55-6.81) | ＜0.0001 |

Note: HR: Hazard Ratio;

Data in parenthesis are 95% confidence intervals;

**Black bold fonts** represent the best performing habitat subregions.

Table S3. Quantitative characteristics of the four habitat subregions in the Validation cohort (n=39)

| Habitat | Volume (%) Mean ± SD | Mean T1CE (a.u.) Mean ± SD | Mean ADC (×10⁻³ mm²/s) Mean ± SD |
| --- | --- | --- | --- |
| Habitat 1 | 32.17 ± 29.70 | 130.38 ± 83.50 | 0.210 ± 0.179 |
| Habitat 2 | 10.87 ± 7.55 | 407.08 ± 197.82 | 2.351 ± 0.105 |
| Habitat 3 | 18.69 ± 19.17 | 782.46 ± 178.84 | 0.757 ± 0.153 |
| Habitat 4 | 38.28 ± 25.27 | 256.27 ± 72.95 | 1.048 ± 0.092 |

Table S4. Prognostic performance of clinical, conventional radiomics and high-risk habitat models(Training cohort).

| Model | Training chort | |
| --- | --- | --- |
|  | C-index | p-value |
| Clinical Model | 0.841 | 0.005 |
| Conventional Radiomics Model | 0.897 | 0.006 |
| High-Risk Habitat Model | 0.933 | 0.001 |
